# The Association between Broiler Litter Microbiota and the Supplementation of *Bacillus* Probiotics in a Leaky Gut Model

**DOI:** 10.3390/ani14121758

**Published:** 2024-06-11

**Authors:** Darwin Horyanto, Yadav S. Bajagai, Juhani von Hellens, Xiaojing Chen, Thi Thu Hao Van, Mark W. Dunlop, Dragana Stanley

**Affiliations:** 1 Institute for Future Farming Systems, Central Queensland University, Rockhampton, QLD 4701, Australia; darwin.horyanto@bioproton.com (D.H.); y.sharmabajagai@cqu.edu.au (Y.S.B.); 2 Bioproton Pty Ltd., Brisbane, QLD 4110, Australia; juhani@bioproton.com (J.v.H.);; 3 School of Science, RMIT University, Melbourne, VIC 3083, Australia; thithuhao.van@rmit.edu.au; 4 Department of Agriculture and Fisheries, Queensland Government, Toowoomba, QLD 4350, Australia; mark.dunlop@daf.qld.gov.au

**Keywords:** litter, microbiota, probiotics, *Bacillus*, broiler, leaky gut

## Abstract

**Simple Summary:**

The last decade of intensive research on the role and importance of intestinal microbial communities has brought abundant novel information and a high appreciation of microbiome contribution to medical and veterinary research that stretches beyond probiotic and pathogenic effects. We can now investigate the complex microbial community inhabiting major host cavities and colonising organs. In poultry research, it is common to collect cloacal swabs or faecal droppings for non-invasive sample collection or caecum and other intestinal origins to investigate their specific microbial communities. Litter samples are less frequently collected for microbial community investigation despite them being in close contact with birds and playing a significant role in microbiota colonisation, development, and maturation. Here, we explored the microbiota of litter and compared them with selected gut sections and bird health status.

**Abstract:**

Probiotics provided from hatch have a major influence on microbiota development, and together with environmental and bedding microbiota, shape the microbial community of the litter. We investigated the influence of probiotic supplementation and a leaky gut challenge induced using dexamethasone (DEX) on the litter microbial community and litter parameters. The probiotic product was a mix of three *Bacillus amyloliquefaciens* strains. The litter microbiota were compared to the microbial communities from other gut sections. The litter samples had higher microbial diversity compared to the caecum, gizzard, jejunum, and jejunal mucosa. The high similarity between the litter phylum-level microbiota and gizzard microbiota detected in our study could be a consequence of ingested feed and litter passing through the gizzard. Moreover, the litter microbial community is fundamentally distinct from the intestinal microbiota, as evidenced by the number of genera present in the litter but absent from all the intestinal sections and vice versa. Furthermore, LEfSe analysis identified distinct microbial taxa across different groups, with specific genera associated with different treatments. In terms of litter quality, the birds in the DEX groups had a significantly higher moisture content, indicating successful leaky gut challenge, while probiotic supplementation did not significantly affect the moisture levels. These findings provide comprehensive insights into the distinct microbiota characteristics of litter.

## 1. Introduction

The gastrointestinal tract (GIT) of poultry encounters exogenous microorganisms immediately post-hatch, creating a conducive environment for a complex microbial community, including commensal, symbiotic, and pathogenic organisms [1]. The journey of bacterial succession and colonisation in domestic broilers may start with incubated eggs, either through close contact with hens or via contact with commercial incubators within 21 days of embryonic development [2], and the environment shortly post-hatching [3]. The early colonisation of exogenous microorganisms provides a baseline environment for creating a stable and diverse microbiota in poultry. Firstly, the gut of chickens is colonised by facultative aerobes and substituted by anaerobes [4]. Then, prolific oxygen consumption by aerobic bacteria promotes the conditions for subsequent colonisation by anaerobic bacteria in the gut ecosystem [5]. The environmental influence in this stage is immense, with birds in close contact with litter and faecal droppings, contributing to the microbiota’s sharing and uniformity. The colonisation process is rapid, followed by maturation, which continues throughout the short production cycle of commercial broilers at approximately 5–6 weeks, while chickens should become sexually mature at 18 weeks. Thus, our insights into the broiler intestinal microbiota are limited to the very early life stages, which are still essential for broiler health and performance.

As the broiler host grows, the microbiota experience significant diversification while the immune system gradually matures over two weeks [6] and stabilise into a steady yet dynamically evolving state. Poultry have a short GIT and a short feed transit time (4–5 h in 29-day-old broilers) [7]. This anatomical distinction selects specialised fast-growing gut microbiota, characterised by extensive interactions among gut microbes and host and dietary components. The interaction of large and complex communities of bacteria plays a significant part in host health, nutrition and physiology [1], such as the production of short-chain fatty acids (SCFAs), essential amino acids and vitamins. The microbial community assists in immune system development and gut homeostasis, provides protection against pathogens, and improves growth performance [8]. 

Studies in broiler gut microbiota examine the microbial composition of specific GIT sections, such as the caecum and ileum, as well as in excreta, litter and poultry house dust [7]. Previous molecular studies on litter microbiota for population-level surveillance of bacteria, viruses, and protozoa in commercial broilers have shown that specific microorganisms of significance can be detected in samples collected from broiler houses even when they are present at low levels [9,10,11]. This indicates that such samples may serve as a viable option for population-level pathogen monitoring programs and potentially for assessing gut microbiota as well. Thus, litter sampling could contribute considerably to poultry management and health status monitoring programs as molecular diagnostics become more viable [12].

Throughout the broiler growth cycle, birds continuously practice coprophagy as well as pecking, and ingest litter particles [13]. Litter generally comprises chicken excreta and bedding material, which serves as bedding and a substrate for microbial activity and waste absorption [14]. Overgrowth of microbial communities could occur because of excess non-digested materials in the hindgut [4]. This disrupts the homeostasis of the gut microbiota and host, producing metabolic, pathogenic and other diseases [15]. The microbial composition present in broiler litter greatly impacts the broiler gut microbiota because of continuous ingestion and contact with microorganisms [16]. Litter accumulates and harbours a complex microbial community partially originating from the gut microbiota, potentially impacting litter reuse for several growth cycles, a common management practice among some poultry producers aimed at cutting production costs and addressing the challenges associated with litter disposal [17]. This practice has implications for the microbial community present in the litter, which can subsequently impact the broiler gut microbiota. For example, a study by Cressman et al. (2010) observed a higher proportion of environmental bacteria harboured in fresh litter, whereas reused litter contained more bacteria of intestinal origin [18]. The same study also demonstrated that the ileal mucosa-associated bacteria of broiler chickens reared on fresh litter were primarily composed of *Lactobacillus* spp. In contrast, birds reared on reused litter were dominated by a group of unclassified *Clostridiales* bacteria [18]. Moreover, the microorganisms present in the reused litter can act as a competitive exclusion culture, delaying the colonisation of *Clostridium perfringens* in the broiler ileal mucosa during the early post-hatch period [19]. However, reused litter may also harbour pathogenic and opportunistic microorganisms from previous batches [20].

Poultry house dust could potentially serve as a non-invasive sample for monitoring bird microbiota at the population level [7]. In poultry dust, most on-farm airborne particulate matter (PM) originates from feathers and manure, where manure contributes most to coarse PM. Feed has a negligible contribution (<16%), and wood shavings contribute <34% [21]. Based on a microbiota study by O’Brien et al. (2016), poultry dust is predominantly composed of bacteria (64–67%) and a minor portion of avian, human and feed DNA (<2%) [22]. Moreover, a study by Yan et al. (2019) [23] pointed out ~99% of the operational taxonomic units (OTUs) in caecal samples were present in excreta, and ~87% of the OTUs in ileal samples were present in excreta, showing the succession patterns of gut microbiota species. Another study also described a correlation between seasonal variations in the litter microbiota and seasonal variations in chicken productivity [13]. This means a succession of microorganisms is observed post-initial gut colonisation, and the population structure of the microbiota increases as birds grow, as they eventually mature and stabilise. This process is intensive in the first three weeks [3,24]. Recognising the physiochemical properties of litter (pH, moisture) is important, particularly with the high stocking densities of commercial broiler houses. The moisture content of litter has been associated with microbial activity, ammonia and odour emissions, as well as bird health and wellbeing [25]. For example, changes in pH, moisture, and organic and inorganic content might impact both biotic and abiotic environments and hence the microbiota [18]. PM is important and can compromise bird and human health, as well as the environment. This correlation proves that a constant exchange of microorganisms exists in the environment and the gut microbiota of broilers and highlights the importance of litter as a reservoir of microbial diversity.

Probiotics, such as *Lactobacillus* spp. and *Bacillus* spp., have been suggested as promising alternatives to antibiotic growth promoters (AGPs) [26,27,28]. The present study investigates the litter microbiota of broilers with and without a *Bacillus*-based probiotic product containing a mix of *B. amyloliquefaciens* strains in a leaky gut model. As the litter microbiota play a crucial role in shaping the structure and dynamics of the gut microbiota and subsequently influencing health and performance, it is important to study the litter microbiota, which is often neglected in poultry microbiota studies. Moreover, good gut health promotes digestibility and decreases the production of NH_3_ and other gases in the poultry housing environment [29], which may pose a danger to farm workers and birds. The birds in the present study were subjected to a gut barrier dysfunction model induced by dexamethasone (DEX) to investigate the role of DEX challenge and *Bacillus* supplementation on litter quality and microbial community.

## 2. Materials and Methods

### 2.1. Sample Collection

The Central Queensland University’s Animal Ethics Committee approved the present study (approval number 0000023123). Two hundred and fifty-six day-old Ross 308 broiler chicks hatched in the same research facility were randomly assigned to 32 pens with eight birds per pen in a completely randomised 2 × 2 design. Each pen was covered with 70 L of fresh wood shavings, 5 cm deep. The broiler house and the equipment, including the pens, were comprehensively cleaned before the hatchlings were placed in the pens to minimise the presence of microbes from previous studies. The supplemented probiotic (Natupro NG, Bioproton, Acacia Ridge, Brisbane, Australia) was a combination of 6 × 10^8^ CFU/g *Bacillus amyloliquefaciens* strains (BPR-11, BPR-16, and BPR-17) supplemented at 500 g/t of feed mixed into a commercially available antimicrobial-free chicken starter diet (Red Hen, Laukee Mills, Australia), formulated to meet or exceed the National Research Council guidelines for broiler chickens. The feed contained wheat, triticale, barley, oats, peas, lupins, lentils, beans, soyabean, canola, sunflower, meat meal, fish meal, blood meal, fat, limestone, di-calcium phosphate, potassium carbonate, sodium bicarbonate, salt, bentonite, lysine, methionine, threonine, tryptophan, isoleucine and valine. Vitamins A, D3, E, K, B1, B2, B3, B5, B6, B7, B9 and B12, choline and the minerals calcium, phosphorus, potassium, sodium, chloride, cobalt, copper, iodine, iron, manganese, molybdenum, selenium and zinc were supplemented. The mixing of probiotic supplement was carried out using an electric mixer to achieve homogeneous supplementation, as in Table 1. The feed was mixed weekly using an electric mixer and stored in a cool, dry place (cold room).

A commercial basal diet (BD) containing all the essential nutrients but free of antibiotics or coccidiostat was provided, and depending on the probiotic and DEX supplementation, was labelled as one of four treatments. We used the letter P for probiotic, D for DEX and C for the unsupplemented control; thus, the abbreviation C = the BD only, the non-supplemented control; CD = the BD with DEX supplementation; P = the BD supplemented with the probiotic and PD was the BD containing the probiotic and DEX supplementation. The probiotic was added in powder form to the BD from day one and was maintained throughout the study period.

On days 28–35 (week 5), birds in the DEX groups were supplemented with DEX at a concentration of 0.6 mg/kg, inducing gut barrier dysfunction [30]. The stock solution of DEX was prepared daily by dissolving DEX (Sigma-Aldrich, St. Louis, MO, USA) in 1 mL of 70% ethanol, which was diluted with 50 mL of water per 20 kg of feed. The DEX solution was sprayed into the feed while mixing it in an electric feed mixer. The DEX-supplemented feed was prepared daily. Feed and water were provided ad libitum. 

Litter samples were collected on day 35 after the DEX leaky gut challenge. Litter from each pen was sampled from five collection points, mixed and stored in sterile plastic containers. The four samples were collected from the centre of each of the four quadrants, and one sample was selected from the middle of the pen. The middle of the pen was near the feeder, and one of the four quadrant samples was adjacent to the drinker. The sample was taken as a 3 cm core, thus sampling only the top layer of litter in direct contact with the birds. This process was carried out three times per pen, producing three replicates per pen (*n* = 96 samples). The samples were stored at −80 °C before being used for DNA sequencing and moisture content and pH evaluation. 

In addition to the litter, the microbiota samples collected in this study included gizzard, caecum, jejunum and jejunal swabs. The microbial communities of each gut section and animal performance data have been published separately by Horyanto et al. [31].

### 2.2. DNA Extraction and Sequencing

Litter DNA (*n* = 96) was extracted using DNA mini spin columns (Enzymax LLC, CAT# EZC101, Lexington, KY, USA) in accordance with the lysis protocol suggested by Yu and Morrison (2004) [32]. The concentration of DNA was measured using a NanoDrop™ One Spectrophotometer (Thermo Fisher Scientific, Wilmington, NC, USA). Subsequently, a DNA sequencing library was generated through amplification of the V3-V4 regions of the 16S rRNA gene using the primer pairs pro341F (5′-CCTACGGGNBGCASCAG-3′) and pro805R (5′-GACTACNVGGGTATCTAATCC-3′), incorporating index, heterogeneity spacer, and Illumina sequencing linkers [33]. Library purification was performed using AMPure XP Kits (Beckman Coulter, Brea, CA, USA) before sequencing on the Illumina Miseq platform in paired-end configuration (2 × 250 bp).

### 2.3. Litter Moisture Content and pH

The litter samples were defrosted, and a combination of 35–55 g of content from three jars per pen was collected and homogenised, producing 32 experimental samples. The samples were placed in an aluminium foil pan, dried at 65 °C for 48 h, and weighed as described by Elias et al. (2020) [34]. Their moisture content was calculated as a percentage. Ten grams of the litter sample was diluted with 100 mL of distilled water and placed in a mechanical shaker for two hours. The supernatant was filtered, and the pH was measured using an Orion 2 Star pH meter (Thermo Fisher Scientific, Wilmington, NC, USA) and the manufacturer-recommended buffers.

### 2.4. Bioinformatics

Raw sequences were demultiplexed using Cutadapt [35], and better-quality reads meeting a minimum Phred score of 25 across a 200 nt length were selected for analysis using Quantitative Insights into Microbial Ecology 2 (QIIME2) [36]. Quality filtering, denoising, and chimera exclusion were performed via the Dada2 [37] plugin, and taxonomic assignments were made using the SILVA v 138.1 database [38]. Further downstream statistical analysis and visualisation were completed by using various R (v4.2.2) packages, including Phyloseq v1.42.0 [39], Phylosmith v1.0.7 [40], Vegan v2.6-4 [41] and Microeco v1.3.0 [42].

## 3. Results

### 3.1. Animal Performance

The broiler performance data, including their body weight (BW), average daily gain (ADG), average daily feed intake (ADFI), and feed conversion ratio (FCR) before and after the DEX periods, are provided in Supplementary Figures S1 and S2 [31]. There was no significant difference in the broiler BW, ADG, ADFI or FCRs between the control and probiotic groups before DEX supplementation, except on day 21 (*p* < 0.05). During the DEX period, a significant difference was observed in all the production parameters between the non-DEX and DEX-challenged groups (*p* < 0.05). A similar performance was evident in both the probiotic-supplemented and non-probiotic groups.

### 3.2. Litter Microbiota Richness and Diversity

**Figure 1:**
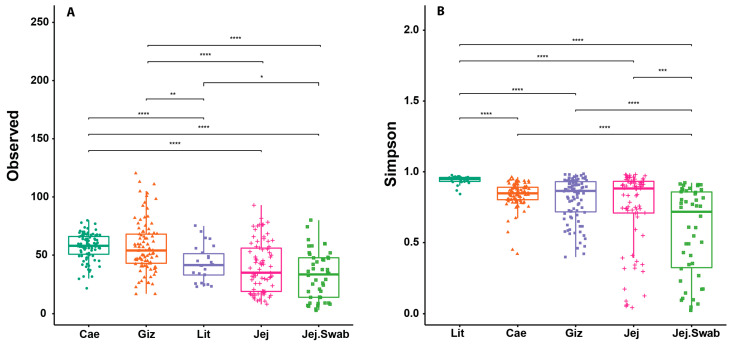
Litter sample alpha diversity presented as Observed Species (**A**) and Simpson metrics (**B**). Lit = litter, Giz = gizzard, Jej = jejunum lumen, Jej.Swab = jejunum mucosal swabs. (* = *p* ≤ 0.05; ** = *p* ≤ 0.01; *** = *p* ≤ 0.001; **** = *p* ≤ 0.0001).

The litter microbiota exhibited distinct characteristics compared to the intestinal microbiota samples (Figure 1). The microbiota richness in litter samples, as measured with Observed Species, is significantly lowerthan that in the caecum and gizzard and significantly higher than the richness in the jejunal swab. However, the litter samples had significantly higher diversity, measured using the Simpson index, than any other analysed gut section microbiota.

No significant variations in the overall microbial richness or diversity were driven by either the DEX or probiotic supplementation.

### 3.3. Litter Microbial Community

**Figure 2:**
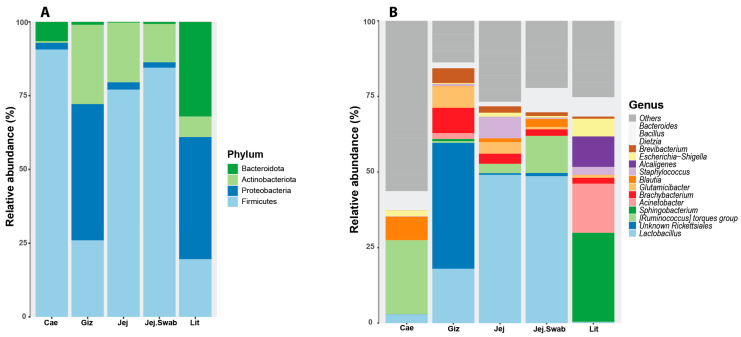
Litter sample microbial community membership compared to other gut sections at phylum (**A**) and genus (**B**) levels.

The phylum-level litter microbiota showed similarities with the gizzard regarding a high abundance of *Firmicutes* and *Proteobacteria* (Figure 2), but it shared the same phylum-level membership with the other gut sections. On the other hand, strong dissimilarities between the litter and the other gut sections’ microbiota became apparent at the genus level. Some dominant genera present in the litter samples detected using 16S amplicon sequencing included *Sphingobacterium*, *Acinetobacter*, *Brachybacterium*, *Glutamicibacter*, *Staphylococcus*, *Alcaligenes*, *Escherichia-Shigella* and *Brevibacterium*. The known genera present in the gut but not in the litter and conversely the known genera present only in the litter but not in any gut sections detected using 16S amplicon sequencing are shown in Supplementary Tables S1 and S2, respectively.

### 3.4. Beta Diversity

**Figure 3:**
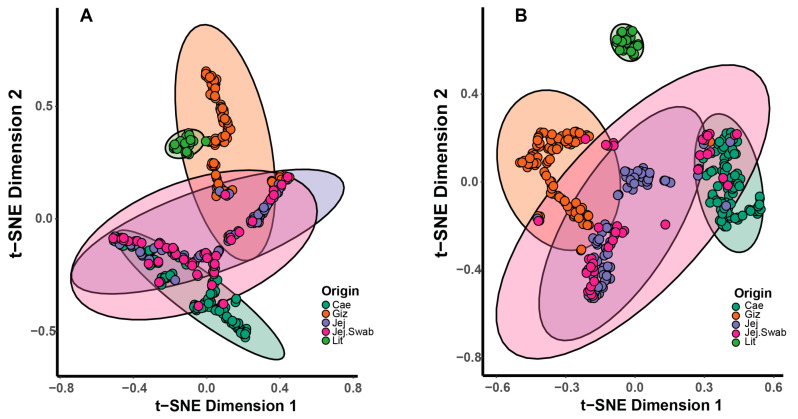
The t-SNE plot demonstrates the grouping of phylum-level (**A**) and genus-level (**B**) microbiota profiles based on individual origins. Ellipses represent 95% confidence intervals. Lit = litter, Giz = gizzard, Jej = jejunum lumen, Jej.Swab = jejunum mucosal swabs.

Overall, non-metric multidimensional scaling (NMDS), principal coordinate analysis (PCoA) and stochastic neighbour embedding (t-SNE) plots showed a clear separation by sample origin, as demonstrated using t-SNE in Figure 3. The litter microbiota samples were highly uniform, clustered closely and separated from the other origins at the genus level, but they were close to those from the gizzard at the phylum level, which agrees with the data presented in Figure 2.

PERMANOVA of the litter microbiota showed significant differences introduced by the DEX challenge based on both the Unweighted (*p* = 0.001) and Weighted UniFrac distances (*p* < 0.022). The treatments, including C, CD, P and PD, were only significant using Unweighted UniFrac (*p* = 0.010) (Table 2). Moreover, paired MANOVA presented significant differences between C and CD (*p* = 0.007), CD and P (*p* = 0.001) and P and PD (*p* = 0.021) using the Unweighted UniFrac distances. However, no significant differences were observed using the Weighted UniFrac distance (Table 3).

Paired MANOVA performed between the gut sections and litter microbiota showed highly significant (*p* < 0.001) differences in all the comparisons between gut sections and between the litter and gut sections using either Weighted or Unweighted UniFrac, as expected. 

**Figure 4:**
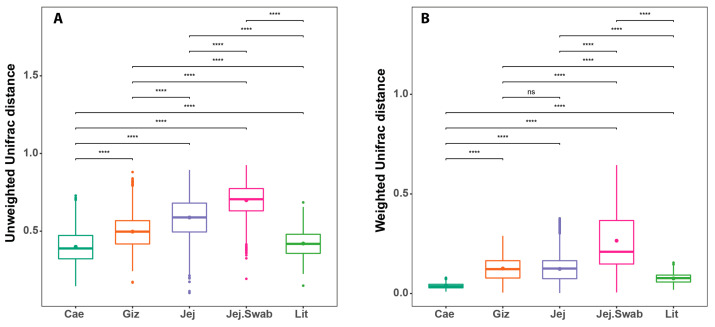
Within-group beta diversity measures show that litter samples are more similar to one another than most other gut sections by Unweighted UniFrac (**A**) and Weighted UniFrac distance (**B**). (ns = *p* > 0.05; **** = *p* ≤ 0.0001).

Further comparisons of the beta diversity within each sampled origin revealed highly significant differences in the sample distribution using Unweighted UniFrac (Figure 4A) and Weighted UniFrac (Figure 4B), indicating high within-group sample-to-sample microbiota differences based on microbiota membership and abundance. The gizzard and jejunum had similar sample-to-sample distances based on weighted UniFrac. 

The litter samples were highly uniform (smaller sample-to-sample distance) compared to the gizzard, jejunum and jejunal swabs samples based on both Weighted and Unweighted UniFrac. The within-sample origin similarity (lower sample-to-sample distance) between the litter samples was most comparable to that represented by the smaller distances (higher similarities) between the caecum samples.

### 3.5. Effects of Treatment on Litter Microbial Taxa

**Figure 5:**
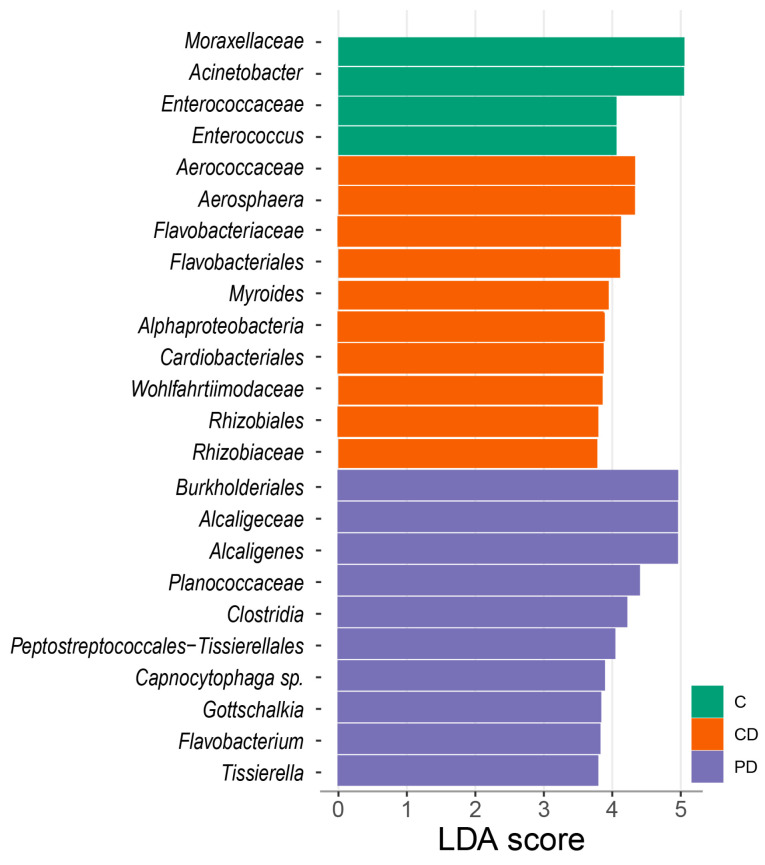
LEfSe analysis presents taxa at all taxonomic levels in litter microbiota (*p* < 0.05 and LDA > 3.5). LDA = linear discriminatory analysis; LEfSe = LDA effect size.

The linear discriminant analysis (LDA) effect size (LEfSe) analysis in Figure 5 presents the distinct microbial taxa across different groups. The litter microbiota were characterised by a higher abundance of *Moraxellaceae*, *Acinetobacter*, *Enterococcaceae* and *Enterococcus* in the C group and *Aerococcaceae*, *Aerosphaera*, *Flavobacteriaceae*, *Flavobacteriales*, *Myroides*, *Alphaproteobacteria*, *Cardiobacteriales*, *Wohlfahrtiimodaceae*, *Rhizobiales* and *Rhizobiaceae* in the CD group. The PD group was represented by *Burkholderiales*, *Alcaligeceae*, *Alcaligenes*, *Planococcaceae*, *Clostridia*, *Peptostreptococcales*-*Tissierellales*, *Capnocytophaga* spp., *Gottschalkia*, *Flavobacterium* and *Tissierella* (Figure 5). No genera were associated with the P group. The genera associated with the supplementation of DEX and the probiotic according to the LEfSe analysis (LDA > 3.5) are presented in Supplementary Figures S3 and S4.

### 3.6. Litter Quality

**Figure 6:**
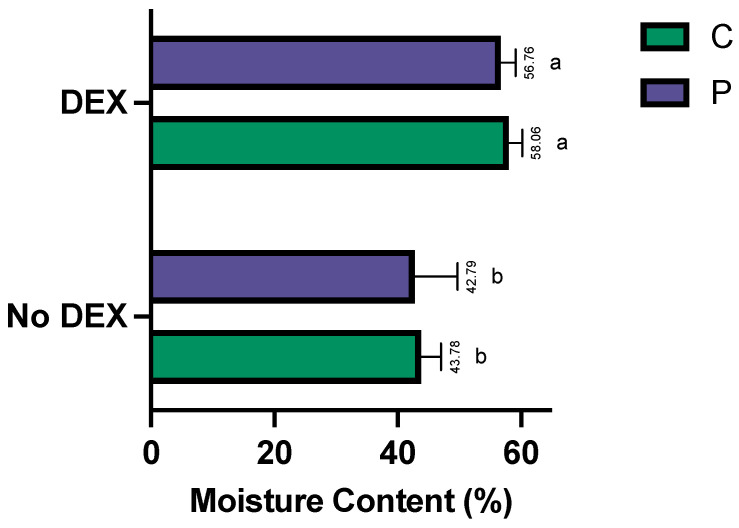
The average moisture content values expressed as percentages (%) and standard error (SE) of C, P, CD and PD groups. ^a,b^ Statistically significant = *p* < 0.05.

The moisture content is presented in Figure 6. The litter in the DEX groups had a significantly higher moisture content than that in the non-challenged groups, indicating that the diarrhea leaky gut challenge was successful (*p* < 0.05). Moreover, there were no significant differences in the moisture levels due to probiotic supplementation (*p* > 0.05). There were no significant differences in the litter pH between the four treatments (*p* > 0.05) (Supplementary Figure S5: Litter pH in C, CD, P and PD groups).

## 4. Discussion

As expected, the moisture content was significantly higher in the leaky gut DEX-challenged groups (CD and PD, *p* < 0.05). The glucocorticoid DEX induces gut inflammation and promotes significant alterations in the intestinal structure, stimulating a condition known as leaky gut, as documented by previous studies [43,44]. This disruption in the gut barrier contributes to decreased digestibility, water loss, and diarrhea, consequently increasing the moisture levels in the litter [45]. Moreover, a leaky gut is associated with the movement of gut bacteria, microbial compounds and/or antigens, prompting a systemic immune response. The systemic inflammation generated by DEX demands energy, decreasing bird production performance [46]. 

The poultry GIT harbours complex microbiota, and the 16S amplicon methodology exclusively investigates a major portion of the bacterial community, which can profoundly impact broiler performance. The absence of significant variations in the alpha diversity indices (Figure 1) suggests DEX and probiotic supplementation exert minimal impacts on overall microbial diversity. A more prominent distinction is seen in beta diversity. The litter in the t-SNE plots is closely clustered and separated from the other sample origins at the genus level but is proximate to the gizzard at the phylum level. The phylum-level litter microbiota showed a high abundance of *Firmicutes* and *Proteobacteria*. This could be a result of ingested feed and litter microbiota passing through the gizzard. The observed variations in microbial diversity between the litter and intestinal samples highlight the importance of considering environmental context [47]. The litter microbial profile is influenced by factors such as the substrate composition and environmental conditions, which exhibit distinct microbial dynamics compared to the gut, which is mainly shaped by environment (moisture content, pH), host physiology and diet [48]. 

Litter containing 43–67% moisture has a greater bacterial abundance compared to that with 10–25% moisture content [49]. This confirms our observation in Figure 1, where higher moisture leads to higher diversity in the litter samples as measured using the Simpson diversity index. A litter moisture content surpassing 40% has been associated with poor digestion and changes in digesta viscosity and protein levels [50] and is often an indicator of diarrhea. The high moisture content, approaching 60%, in the DEX groups provides ideal conditions for anaerobes, such as *Clostridia* and *Peptostreptococcales*-*Tissierellales*, whereas a ~40% moisture content in the non-DEX groups may have been slightly aerobic, which is ideal for aerobes, such as *Moraxellaceae* and *Acinetobacter*. Another study observed something similar, where *Clostridia* is predominant in fresh litter and ileal mucosa microbiota [18]. Hence, a high moisture content or “wet litter” is a safe haven for pathogens (*C. perfringens*, *Escherichia coli*), which can increase the incidence of *necrotic enteritis* (NE) and secondary health problems, such as footpad dermatitis, ammonia proliferation and poor overall welfare and performance in broilers [49].

No genera were significantly associated with probiotic supplementation, according to the LEfSe analysis, when observing all four treatments. The litter microbiota in the DEX groups were characterised by *Acinetobacter*, *Enterococcaceae*, *Enterococcus*, *Myroides*, *Burkholderiales*, *Alcaligeceae*, *Alcaligenes*, *Clostridia*, *Peptostreptococcales*-*Tissierellales*, *Capnocytophaga* spp., *Gottschalkia*, *Flavobacterium* and *Tissierella*. These bacteria can cause chronic diseases in poultry. *Enterococcus faecium* is deemed a member of the gut microbiota in both humans and poultry. *Acinetobacter*, part of the *Moraxellaceae* family, is a strictly aerobic microorganism that mostly occurs in spoiled meats and is considered a mild spoiler of meat and dairy products because of its poor proteolytic activity but good lipolytic activity [51]. 

Members of the *Myroides* genus associated with the CD group originate from the family *Flavobacteriaceae* and are considered opportunistic pathogens in poultry. Moreover, some species of the genus *Flavobacterium* associated with the PD group are related to poultry diseases and respiratory infections [52]. The order *Burkholderiales* includes a diverse group of bacteria, but a study by Campos et al. (2022) [53] pointed out that *Burkholderiales* is more abundant in birds infected by *Eimeria tenella*. 

Some species of *Alcaligeceae* like *Bordetella avium* are often present in soil and water environments and are considered zoonotic, contagious and a common cause of a disease called bordetellosis in birds [9,54]. Some clostridia are considered very important agents of enteric disease in poultry. For example, the incidence of *C. perfringens*-associated NE in poultry has increased significantly in countries discontinuing the use of AGPs [55]. The growth, survival and spread of both clinical and subclinical NE, a major disease in the broiler industry, is promoted by a high moisture content in the litter [49]. These anaerobic microorganisms are also involved in cattle disease pathogenesis and act as secondary opportunistic pathogens [56]. *Capnocytophaga* spp. are commonly seen in the oral cavities of dogs and cats [57], whereas *Gottschalkia* are pathogens and were also present in layer manure samples from different-scale farms in a study conducted by Wang and Chai (2022) [58].

*Planococcaceae*, which are selected as characteristic of PD birds, are common inhabitants of poultry GITs. A study by Abdel-Kafy et al. (2022) identified *Planococcaceae* in the mucosa of high-weight birds [59]. Moreover, Mon et al. (2020) stated *Planococcaceae* and *Lactobacillaceae* are often highly co-abundant [60]. *Aerococcaceae* were also present in ileal and caecal content samples in a study by Bindari et al. (2021) [7]. *Alphaproteobacteria*, a class of *Proteobacteria*, was seen in the CD birds. *Proteobacteria* is a phylum commonly present in younger birds before the microbial succession moves towards high domination by *Firmicutes* [4]. Another study stated that the highly pathogenic phylum *Proteobacteria* is a predominant phylum in cloacae because of its oxygen tolerance [61]. The order *Cardiobacteriales* is closely correlated with *Wohlfahrtiimonas* [62]. *Wohlfahrtiimonas* is isolated from parasitic fly larvae, *Wohlfahrtia magnifica* [63], and could be present in litter via insect vectors. Finally, *Rhizobiales* commonly colonise plants and live in symbiosis, promoting N-cycling. There is minimal information on the family of *Rhizobiaceae* in poultry. One study mentioned that *Rhizobiaceae* were abundant on eggshell surfaces post-hatching [64], but another study correlated *Rhizobiaceae* and a decrease in the FCR [65].

While poultry litter contains microbiota originating from excreta, its microbial community cannot be considered similar to the microbiota from poultry gut sections based on their shared intestinal taxa. Considering that the methodology we use in microbiota studies detects taxa based on the DNA extracted from the source, this methodology cannot distinguish between DNA from live and dead bacteria. It is improbable that strict anaerobes from the intestine would survive and thrive in the aerobic environment of the litter. However, dead excreta bacteria could represent a rich source for the growth of different environmental bacteria. A high level of pathogenic *Proteobacteria* genera in cloacal swabs is likely influenced by litter exposure and *Proteobacteria*’s ability to tolerate oxygen, leading to the overgrowth of poultry pathogens in the litter. At the same time, some of the most beneficial intestinal microbiota used as next-generation probiotics, like *Akkermania* and *Faecalibacterium*, are strict anaerobes. The top layer of the litter (we sampled the top 3 cm core) is exposed to the environment, oxygen, insects and bedding, feed, water and soil microbiota. Thus, controlling the microbial load and moisture content of the litter should be considered in broiler house monitoring programs. 

Controlling environmental microbial counts is a complex process. Most commercial broiler houses have management programs in place, such as providing effective ventilation to simultaneously dry litter and reduce air dustiness [66], as well as using antimicrobial and anti-biofilm agents. Proper ventilation is important, as studies on broiler litter have shown a water activity (A_W_) of <0.84 is suggested as a successful control measure in minimising *Salmonella* populations [67] and microbial activity [25]. This study provides a passive sampling and analysis protocol proposal to assess microbial contamination in litter samples. The litter microbiota also mature and alter with time, and ongoing monitoring may be useful for providing an early warning of potential disease outbreaks.

Co-infection in commercial poultry flocks is likely to occur due to the close interactions between birds and the litter. This is important, as we know birds continuously practice coprophagy and are exposed to the microbiota present in the bedding particles, where pathogens are commonly present [16]. For example, poultry infected by *Histomonas meleagridis* and *E. coli* experienced more severe pathogenesis, a sudden shift in the caecal mucosa-associated microbiota and the colonisation of pathogenic bacteria [68]. Moreover, increased pathogens post-infection or changes in the environmental conditions could decrease the population of beneficial bacteria such as *Lactobacillus*. A study by Oh et al. (2017) observed stabilised and improved gut microbiota post-supplementation of *Bacillus subtilis* against *Salmonella* in hens, which is very positive [69].

As mentioned above, hatchlings can be infected vertically (via hens) or horizontally (via the environment). The ban on AGPs and vaccines whose success depends on specific serovar and host species could alter the microbiota colonisation of mucosal surfaces in young birds [70]. Thus, strategies like proper ventilation and in-feed supplementation of probiotic spores may be successful in minimising water activity and producing competitive exclusion against pathogens [71], modulating the gut microbiota and decreasing the shedding of pathogens, such as *Salmonella*, into broiler environments and meat [70].

## 5. Conclusions

In conclusion, the dexamethasone leaky gut challenge introduced a significant increase in diarrhea, evident from a significant increase in the litter moisture, and resulted in significant shifts in the litter microbiota, more prominent in influencing the presence and absence of species (Unweighted) than their abundance and dominance (Weighted UniFrac). Probiotic supplementation did not alter the litter microbiota. 

Litter samples have very high microbial diversity, significantly higher than that of any other gut origin, including the caecum. However, the litter does not have the highest microbial richness, in this study being slightly behind the caecum and gizzard. Compared to those from the gut sections, the litter microbial samples (sample-to-sample distance) were highly similar to one another. This uniformity can be utilised for both diagnostic and therapeutic purposes. The use of shotgun metagenomic sequencing of the litter could suggest novel biomarkers and open new directions in using litter to improve bird health. 

## Figures and Tables

**Table 1:** Probiotic strains and specification in CFU/g.

Probiotic Strains	Specification
*Bacillus amyloliquefaciens* (BPR-11)	2 × 10^8^ CFU/g
*Bacillus amyloliquefaciens* (BPR-16)	2 × 10^8^ CFU/g
*Bacillus amyloliquefaciens* (BPR-17)	2 × 10^8^ CFU/g

**Table 2:** PERMANOVA of litter by both Weighted and Unweighted UniFrac.

Groups	R2	*p*-Value	Distance
DEX	0.111564	0.001 *	Unweighted UniFrac
Probiotic	0.032266	0.710	Unweighted UniFrac
Treatments	0.172054	0.010 *	Unweighted UniFrac
DEX	0.119781	0.022 *	Weighted UniFrac
Probiotic	0.010500	0.933	Weighted UniFrac
Treatments	0.140897	0.287	Weighted UniFrac

DEX × probiotic interactions were significant (*p* < 0.05) according to Unweighted and Weighted UniFrac. Treatment PERMANOVA compared differences between four groups (C, CD, P and PD). * Statistically significant = *p* < 0.05.

**Table 3:** Paired MANOVA between experimental groups in the litter. There was a significant difference between groups, according to both Weighted and Unweighted UniFrac, each time when comparing the DEX-challenged and unchallenged groups.

Groups	R2	*p* (>F)	Distance
C vs. CD	0.153235	0.007 *	Unweighted UniFrac
C vs. P	0.079020	0.563	Unweighted UniFrac
C vs. PD	0.123330	0.069	Unweighted UniFrac
CD vs. P	0.168461	0.001 *	Unweighted UniFrac
CD vs. PD	0.056801	0.849	Unweighted UniFrac
P vs. PD	0.137487	0.021 *	Unweighted UniFrac
C vs. CD	0.118515	0.257	Weighted UniFrac
C vs. P	0.021170	0.943	Weighted UniFrac
C vs. PD	0.175991	0.078	Weighted UniFrac
CD vs. P	0.091673	0.289	Weighted UniFrac
CD vs. PD	0.026885	0.878	Weighted UniFrac
P vs. PD	0.142875	0.085	Weighted UniFrac

* Statistically significant = *p* < 0.05.

## Data Availability

The raw sequence data are available from the NCBI SRA database under accession number PRJNA1068165: https://www.ncbi.nlm.nih.gov/bioproject/1068165 (accessed on 26 May 2024).

## Supplementary Materials

The following supporting information can be downloaded at https://www.mdpi.com/article/10.3390/ani14121758/s1. Figure S1: Broiler performance metrics, including body weight (BW), average daily gain (ADG), average daily feed intake (ADFI) and feed conversion ratio (FCR), measured before the supplementation of DEX; Figure S2: Broiler performance metrics, including body weight (BW), average daily gain (ADG), average daily feed intake (ADFI) and feed conversion ratio (FCR), between days 28 and 35 after the DEX period; Figure S3: Genera associated with dexamethasone supplementation according to LEfSe analysis (LDA > 3.5); Figure S4: Genera associated with probiotic supplementation according to LEfSe analysis (LDA > 3.5); Figure S5: Litter pH in C, CD, P and PD groups. No statistically significant differences were observed between the groups (*p* > 0.05); Table S1: Known genera present in the gut but not in the litter detected using 16S amplicon sequencing; Table S2: Known genera present only in the litter but not in any gut sections detected via 16S amplicon sequencing.
