# Supplementary material for: The Association between Broiler Litter Microbiota and the Supplementation of Bacillus Probiotics in a Leaky Gut Model
Source: Animals (Basel). 2024 Jun 11;14(12):1758. doi: 10.3390/ani14121758 (PMC11200599; doi:10.3390/ani14121758)
Supplement: Supplementary file 1 [file animals-14-01758-s001.zip › animals-3053186-supplementary.pdf]

animals

Figure S1: Broiler performance metrics, including body weight (BW), average daily gain (ADG), average daily feed intake (ADFI), and feed conversion ratio (FCR) measured before the supplementation of DEX.

Figure S2: Broiler performance metrics, including body weight (BW), average daily gain (ADG), average daily feed intake (ADFI), and feed conversion ratio (FCR) between days 28 and 35 after the DEX period.

Figure S3: Genera associated with dexamethasone supplementation according to LEfSe analysis (LDA > 3.5).

Figure S4: Genera associated with probiotic supplementation according to LEfSe analysis (LDA > 3.5).

Figure S5: Litter pH in C, CD, P, and PD groups. No statistically significant differences were observed between the groups (P > 0.05).

Table S1: Known genera present in the gut but not in the litter detected by 16S amplicon sequencing.

Table S2: Known genera present only in the litter but not in any guts sections detected via 16S amplicon sequencing.

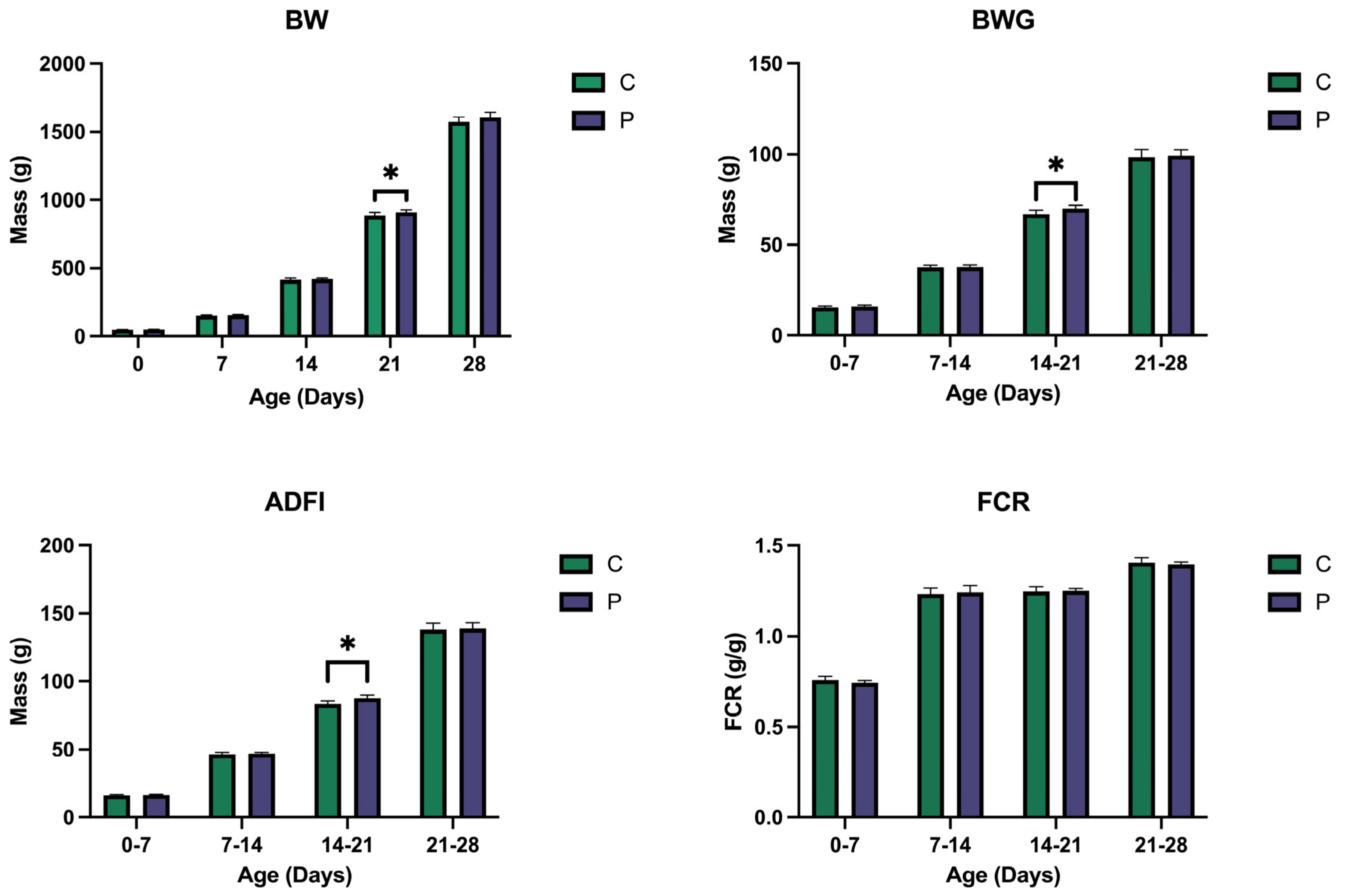

Figure S1: Broiler performance metrics, including body weight (BW), average daily gain (ADG), average daily feed intake (ADFI), and feed conversion ratio (FCR) measured before the supplementation of DEX.

[*] Statistically significant $P < 0.05$.

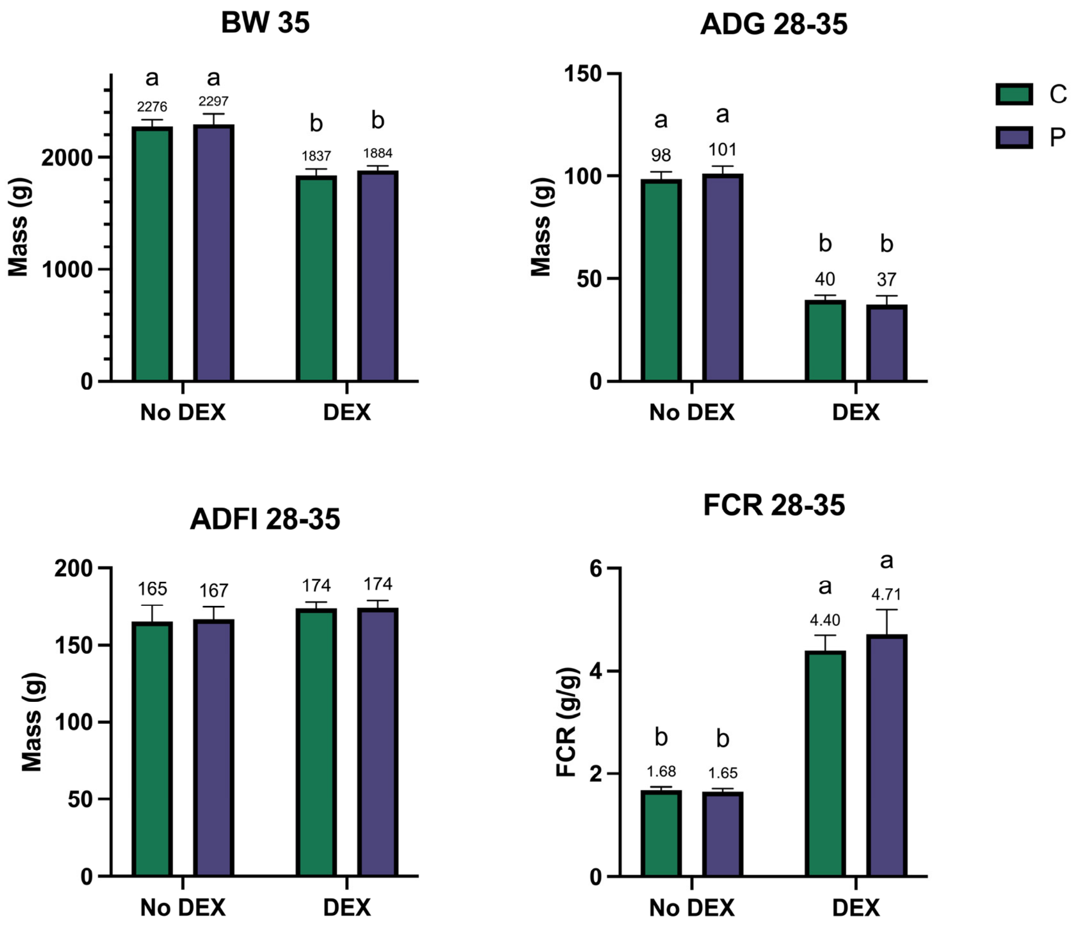

Figure S2: Broiler performance metrics, including body weight (BW), average daily gain (ADG), average daily feed intake (ADFI), and feed conversion ratio (FCR) between days 28 and 35 before and after the DEX period.

[a,b] Different superscript letters indicate statistical significance $P < 0.05$.

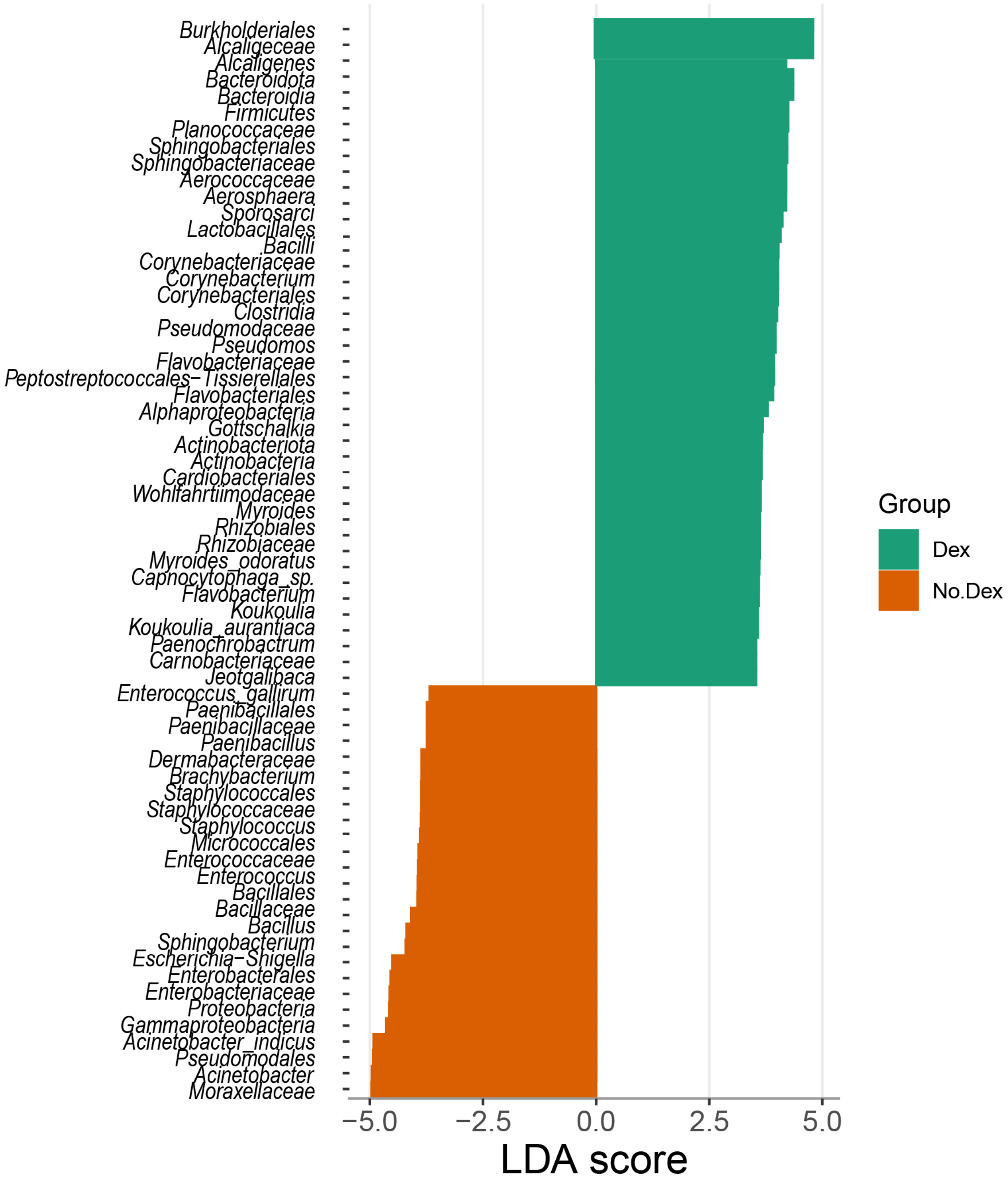

Figure S3: Genera associated with dexamethasone supplementation according to LEfSe analysis (LDA > 3.5).

The analysis compares groups supplemented with DEX = (CD and PD) with non-challenged groups No.DEX = (C and P).

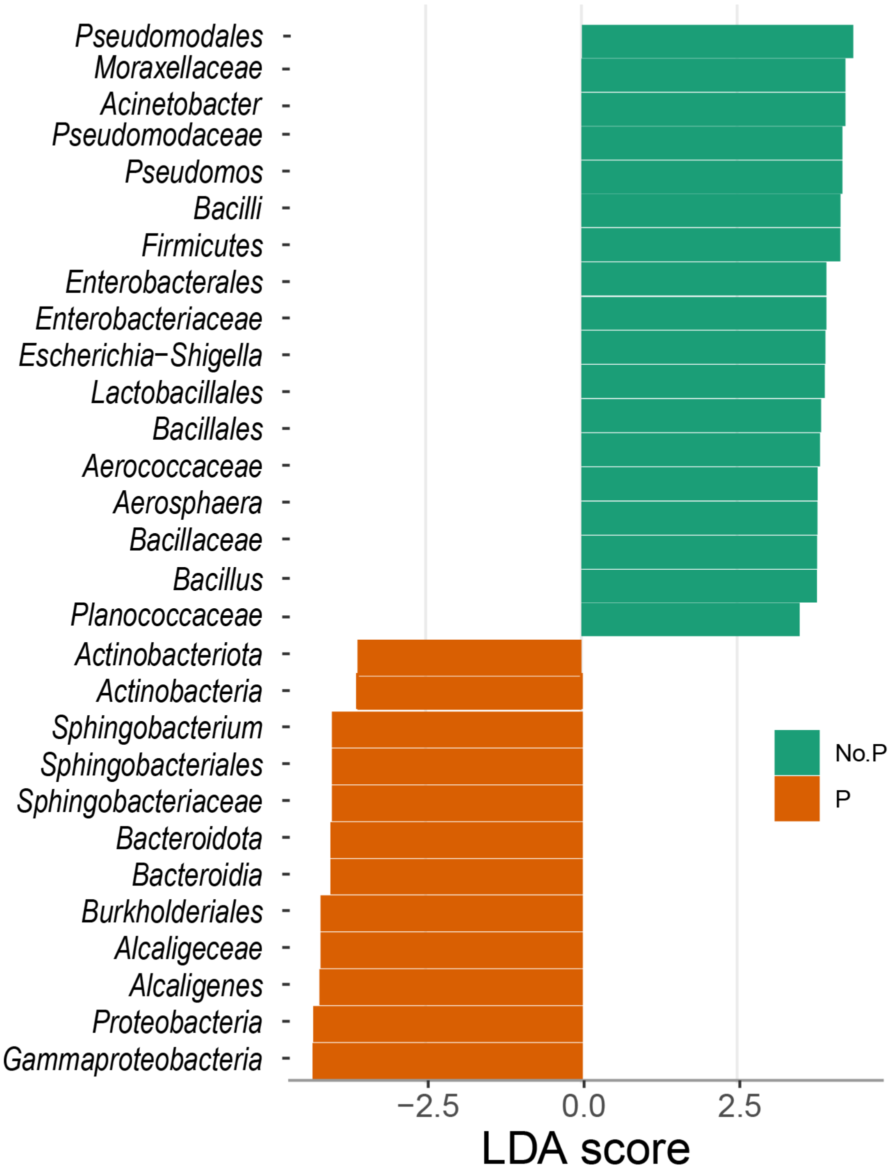

Figure S4: Genera associated with probiotic supplementation according to LEfSe analysis (LDA > 3.5).

The analysis compares groups supplemented with probiotics P = (P and PD) with non-supplemented groups No.P = (C and CD).

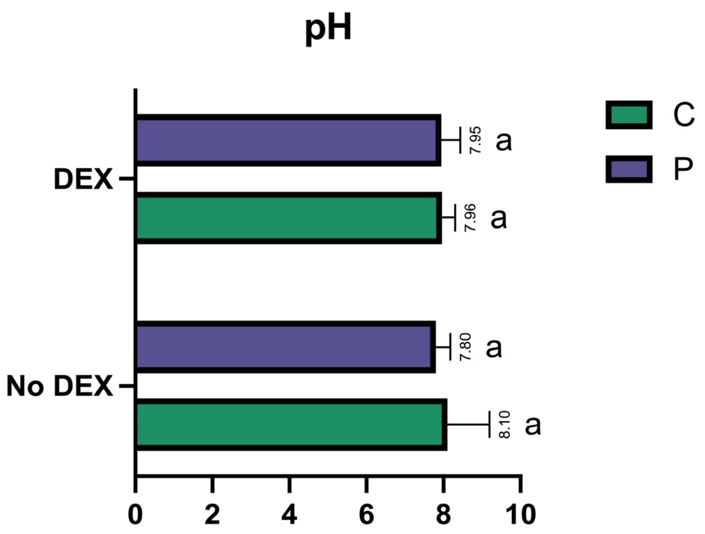

Figure S5: Litter pH in C, CD, P, and PD groups. No statistically significant differences were observed between the groups (P > 0.05).

Litter samples had very comparable pH values with mean pH for C = 8.1; CD = 7.96; P = 7.8 and PD = 7.95.

Table S1: Known genera present in the gut but not in the litter detected by 16S amplicon sequencing

**Genera present in the gut but not in the litter**
Acetobacter
Aerococcus
Aeromicrobium
Aeromonas
Allorhizobium-Neorhizobium-Pararhizobium-Rhizobium
Anaerocolumna
Anaerotruncus
Arthrobacter
Atopostipes
Bifidobacterium
Bordetella
Brevibacillus
Butyricicoccus
Catenibacillus
Cellulosilyticum
Chishuiella
Christensenellaceae
Chryseobacterium
Citricoccus
Clavibacter
Clostridia
Colidextribacter
Coprobacillus
Curtobacterium
Curvibacter
Dechloromonas
DTU014
Eggerthella
Eisenbergiella
Enteroscipio
Exiguobacterium
Ezakiella
Flavonifractor
Fluviicola
Fusobacterium
Georgenia
Gordonibacter
Gulosibacter
Halolactibacillus

Hungatella
Intestinimonas
Kosakonia
Lachnoclostridium
Lactococcus
Lactovum
Leucobacter
Leuconostoc
Massilia
Methylobacterium-Methylorubrum
Methyloversatilis
Monoglobus
Mucilaginibacter
Muribaculaceae
Nakamurella
Nocardiopsis
Novosphingobium
Oscillibacter
Paludicola
Papillibacter
Pedobacter
Pelosinus
Phocea
Pseudoclavibacter
Ralstonia
Rathayibacter
Rhodococcus
Romboutsia
Rothia
Rudanella
Ruminococcus
Saccharibacillus
Saccharopolyspora
Sellimonas
Sphingobium
Sphingomonas
Streptococcus
Terrabacter
Terrisporobacter
TK10
Turicibacter
Tyzzerella

UBA1819
UCG-010

Table S2: Known genera present only in the litter but not in any guts sections detected via 16S amplicon sequencing.

**Genera present only in the litter**
Globicatella
Anaerosalibacter
Herbinix
Defluviitalea
Pusillimonas
Cerasibacillus
Alkaliphilus
